# Investigation of the Sandfly Fauna of Central Arid Areas and Northern Humid Regions of Tunisia, with Morphological and Molecular Identification of the Recently Established Population of *Phlebotomus* (*Larroussius*) *perfiliewi*

**DOI:** 10.3390/insects13111057

**Published:** 2022-11-16

**Authors:** Ifhem Chelbi, Amani Abdi, Jérôme Depaquit, Wasfi Fares, Mohammed Abdo Saghir Abbas, Khalil Dachraoui, Elyes Zhioua

**Affiliations:** 1 Unit of Vector Ecology, Pasteur Institute of Tunis, 13 Place Pasteur BP 74, 1002 Tunis, Tunisia; 2 Université de Reims Champagne-Ardenne, ANSES, SFR Cap Santé, EA4688 ESCAPE–USC «Transmission Vectorielle et Épidémiosurveillance de Maladies Parasitaires (VECPAR)», 51 rue Cognacq-Jay, CEDEX, 51096 Reims, France; 3 Laboratory of Clinical Virology, Pasteur Institute of Tunis, 13 Place Pasteur BP 74, 1002 Tunis, Tunisia

**Keywords:** *Phlebotomus perfiliewi*, morphological identification, molecular identification, bioclimatic areas, zoonotic visceral leishmaniasis

## Abstract

**Simple Summary:**

*Phlebotomus perfiliewi* is one of the main vectors of *Leishmania infantum*, etiologic agent of zoonotic visceral leishmaniasis (ZVL), in the Western Mediterranean basin including Tunisia. *Phlebotomus perfiliewi*, originally endemic only in Northern humid bio-climatic zones, became established in a highly irrigated area of the central arid bioclimatic zone. Therefore, surveillance of sandfly vectors is a cornerstone for the establishment of control strategies. An entomological survey was carried out using light traps in a humid and in a highly irrigated arid bio-climatic zone located in northern and central Tunisia, respectively. Collected sandflies were identified based on morphological criteria, and identification of *Ph. perfiliewi* was confirmed by molecular tools. Results indicated that *Ph. perfiliewi* is the most abundant sandfly species in both sites and it is genetically variable. Thus, surveillance and control of sandflies is highly needed to limit the incidence of sandfly-borne diseases.

**Abstract:**

Based on nucleotide sequences, we re-identified representative samples of *Phlebotomus perfiliewi* originating from two different biogeographical areas of Tunisia, whose populations had previously been identified based on morphological criteria. A partial region of the mitochondrial DNA cytochrome b gene was targeted, and sandfly species was determined by analogy with DNA sequences available in the GenBank database via a BLAST analysis, taking into account the query coverage and percentage identity. The recognized species presents the most substantial homology with the analyzed sequence. The results of the molecular identification showed complete agreement with the morphological identifications, and *Phlebotomus perfiliewi* is genetically variable.

## 1. Introduction

*Phlebotomus perfiliewi* is a proven vector of *Leishmania infantum*, an etiologic agent of zoonotic visceral leishmaniasis (ZVL) in the western Mediterranean basin, including Algeria [1,2], Italy [3], and Tunisia [4,5]. The geographical distribution of *Ph. perfiliewi* covers the southern Mediterranean basin from Morocco, which represents its extreme western range limit [6], to Algeria [7,8] and Tunisia [9,10], and the northern Mediterranean basin from France which represents its extreme western limit in Europe [11], to Italy [12], and Crimea [13]. Prior to 1980, *Ph. perfiliewi* was abundant in humid, sub-humid, and semi-arid biogeographical areas and rare in the south of the Tunisian Ridge [9,10,14]. Therefore, low humidity was considered a limiting factor for the distribution of this sandfly species [15]. Following the intense development of irrigation systems in the arid biogeographical areas in central Tunisia after 1980, *Ph. perfiliewi* became endemic in these highly irrigated areas located in the south of the Tunisian Ridge [4,16]. Subsequently, a stable cycle of *L. infantum* transmission has been established that has led to the emergence of ZVL in these areas [4,5,17,18,19,20,21].

The taxonomic status of *Ph. perfiliewi* is somewhat complex and has been debated. It includes three taxa with a specific or a sub-specific rank: (1) *Ph. perfiliewi sensu stricto* Parrot, 1930 present in North Africa, western Europe, the Balkans, and Crimea, (2) *Ph. galilaeus* Theodor, 1958 in Israel/Palestine and Cyprus, and (3) *Ph. transcaucasicus* Perfiliev, 1937 in the Caucasus and Central Asia [22]. Therefore, in the absence of consensus, these populations are named *Ph. perfiliewi sensu lato* [22]. In Tunisia, as a direct consequence of anthropogenic disturbances due to the development of irrigation systems, the population of *Ph. perfiliewi* might have spread and established in the previously central arid biogeographical areas [4]. Thus, it is of major importance to conduct a faunistic research in previously surveyed areas [4,16]. The present study aims to characterize the sandfly fauna in two distinct bioclimatic regions of Tunisia and confirm the identification of *Ph. perfiliewi* populations with molecular markers. 

## 2. Materials and Methods

### 2.1. Study Sites

**Figure 1:**
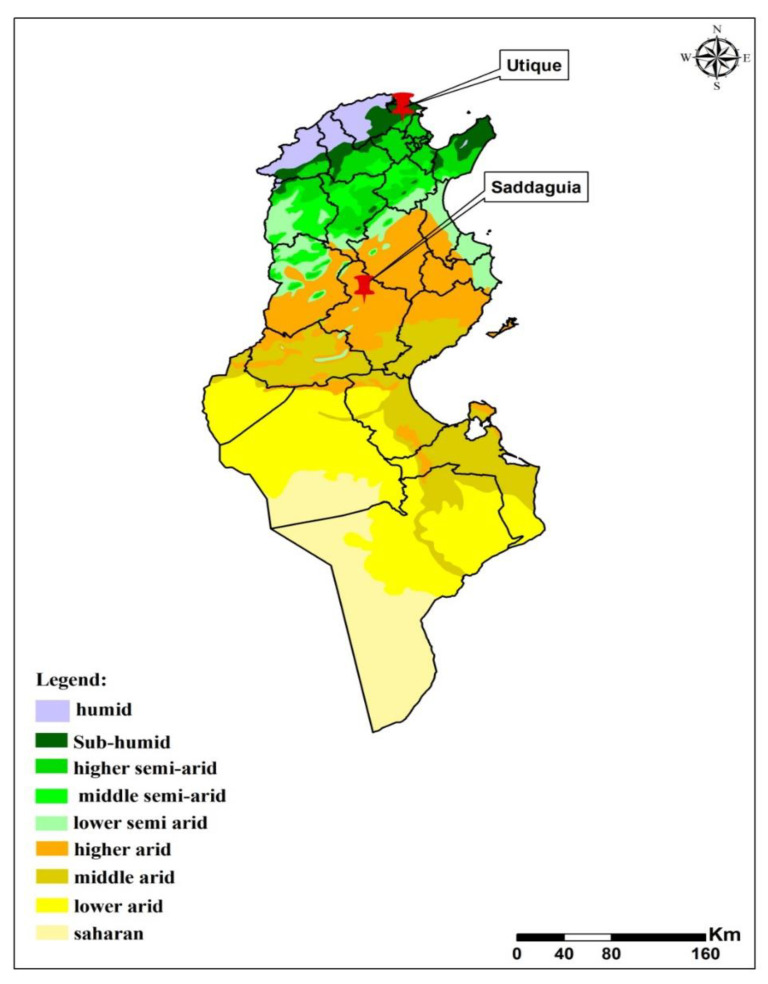
Bioclimatic map of Tunisia showing sandfly sampling sites.

Tunisia spans a wide range of climates, from the rainy (in winter) Mediterranean climate in the north to the Saharan climate in the south. The Tunisian Ridge, running from northeast to southwest for some 220 km, is the climatic boundary between the Mediterranean north and the dry steppe of central Tunisia, delimiting northern and southern Tunisia. An extensive series of plateaus called the High Tell is localized between the north slopes of the Tunisian Ridge and the chains of hills bounding it on the south. Salted areas called chotts separate the Sahara from the central steppe [23] (Figure 1).

### 2.2. Collection of Sandflies 

Sandfly sampling was performed in two rural villages belonging to two bio-climatic zones (Utique 37°08′ N, 7°74′ E, sub-humid; Saddaguia, 34°51′ N, 9°29′ E, arid) (Figure 1). It is important to point out that Saddaguia is a highly irrigated area located in arid central Tunisia [4]. These two villages are not adjacent to mountainsides where houses are located on small farms surrounded by agricultural fields. One house per village was chosen after obtaining oral consent from the landlord. Using only CDC miniature light traps (John W. Hock Company, Gainesville, FL, USA), sandflies were collected inside houses, in peri-domestic areas, and in animal shelters. Traps were placed from dusk to dawn one night per site from June to July 2018 (30 June 2018), corresponding to the first peak of sandfly activity [24,25,26]. A total of 11 CDC light traps were placed in each house corresponding to a total of 22 night traps in both sites. All sandflies were washed in sterile distilled water before dissection. The head and genitalia of each sandfly were removed and mounted on glass slides in Mark André medium [27] for morphological identification to the species level, using the identification keys of Croset et al. [28] and Léger et al. [29]. Special attention was paid to the atypical form of *Ph. perniciosus* females, often misidentified as *Ph. longicuspis* [30,31]. The remaining part of the bodies was placed in PBS at −20 °C for molecular analysis.

### 2.3. Population Structure and Diversity Indices 

We used species richness to measure the numbers of different species of sandflies present in a particular biotope, and we used equality to compare the similarity of the population size of each sandfly species present. When taking into account species richness and relative abundances, the Simpson diversity index was calculated to understand the distribution of individuals among different sandfly species. We used these indices to characterize community structure relative to the rarity or the commonness of the various sandfly species present. The following ecological parameters and indexes were calculated:

Relative abundance (A_r_):

(Number of a sandfly species/Total number of sandflies in the sample) × 100

Species richness (S): number of species in the sample [32]. 

Species diversity was also measured using diversity indexes that simultaneously express the relation between the numbers of species and individuals [32].

Simpson index (I_S_) = 1/∑ pi^2^

_i_pi: Ar/100

I_s_ varies between 1 and S 

I_s_ = 1: dominance of one species, I_s_ = S: species having equal abundances

Shannon index (IH) = I_H_ = −∑pi log 2 pi

Equitability (E): E = I_H_/log2S

E varies between 0 and 1

E = 0: one species dominates, E = 1: species have equal abundance

Data analysis was performed by using XLSTAT to determine ecological parameters and indexes. 

### 2.4. Molecular Analysis 

A total of 20 specimens, morphologically identified as *Ph. perfiliewi,* were selected for molecular analysis with an equal sex ratio, including 5 males and 5 females from each study site (Utique and Saddaguia). DNA extraction was performed using the QIAmp DNA Mini Kit (Qiagen, Hilden, Germany). Confirmation of the taxonomic status of morphologically identified sandfly specimens of *Ph. perfiliewi* was done by amplification of a fragment (450 nucleotides) of the mitochondrial cytochrome b subunit I (Cyt-b) gene [33,34]. It is important to point out that Cyt-b is a very helpful molecular marker able to discriminate two closely related species [35,36].

Forward CB1 primer (5′_TATGTACTACCATGAGGACAAATATC-3′) and CB3-RA reverse primer (5′_GCTATTACTCCYCCTAACTTRTT-3′) used for PCR amplification of the Cyt-b gene fragment were mixed with 5 µL of extracted DNA. The PCR reaction was performed using a Taq DNA recombinant polymerase kit (Invitrogen) in an Applied Biosystems 2700 Thermocycler. The amplification program is as follows: (i) a first denaturation step at 94 °C for 12 min, (ii) 5 cycles starting with denaturation at 94 °C for 30 s, hybridization at 40 °C for 30 s, and elongation at 72 °C for 1 min, (iii) 30 cycles starting with denaturation at 94 °C for 30 s, hybridization at 44 °C for 30 s and elongation at 72 °C for 1 min. The program ended with an elongation step at 72 °C for 10 min. 

Quality of the amplification products was visualized by agarose gel electrophoresis. The products were then purified by the ExoSAP-IT enzymatic purification method based on the association of the exonuclease I (Exo I) with Shrimp Alkaline phosphatase (ShrAP), allowing the preservation of DNA. Purified DNAs were sequenced in both directions using forward and reverse PCR primers (CB1, CB3-RA) and a Big Dye Terminator ready reaction cycle sequencing v3.1 kit (Applied Biosystems). Consensus sequences were deduced from the forward and reverse sequences alignment using CLUSTAL_W 1.4 implemented in MEGA 11 [34]. Taxonomic status was then determined via a search for homology to sequences in the GenBank database using the Blast “Basic Local Alignment Search tool” “http://blast.ncbi.nlm.nih.gov/Blast (accessed on 11 February 2020)”.

The phylogenetic analysis was carried out based on *Ph. perfiliewi* sequences from our work and *Ph. perfiliewi* Cyt-b gene sequences from the GenBank database. Only sequences with at least 90% of coverage were considered. The analysis was performed using MEGA 11 software according to the maximum likelihood method using the Hasegawa-Kishino-Yano model. A total of 1000 bootstrap replications were carried out to evaluate the nodes’ robustness. A sequence of *Ph. perniciosus*, a genetically *Ph. perfiliewi* close species, was used as an outgroup. The intra- and inter-species genetic divergence values were calculated using the p-distance model implemented in MEGA 11 software [37].

## 3. Results

### 3.1. Sandfly Species, Relative Abundance, and Population Structure

A total of 233 and 685 sand flies were collected from Utique and Saddaguia, respectively. Morphological identification of sandflies collected from Utique showed that *Ph. perfiliewi* was the most abundant species (N = 113, 48.5%), followed by *Ph. perniciosus* (N = 92, 39.5%), *Ph. longicuspis* (N = 20, 8.6%), and *Ph. papatasi* (N = 8, 3.4%) (Table 1). 

The population structure of sandflies is characterized by the dominance of two species belonging to the subgenus *Larroussius*. *Phlebotomus perfiliewi* occupied almost half of the sandfly species fauna trapped with a relative abundance of 48%, followed by *Ph. perniciosus* with a relative abundance of 39%. This points to the sympatry of these species within this bio-geographic area. On the other hand, the Simpson and Shannon biodiversity indexes showed that the sandfly fauna is not as diversified because these two values remain lower than the species richness, supported by the low relative abundance of *Ph. longicuspis* and *Ph. papatasi*. The value of Equitability (E) also showed that the guild of the sandfly fauna is not balanced because it is represented by four species that are not equitably distributed.

Morphological identification of sandflies collected from Saddaguia showed that *Ph. perfiliewi* was the most abundant species (N = 295, 43.06%), followed by *Ph. perniciosus* (N = 238, 34.74%), *Ph. longicuspis* (N = 65, 9.48%), and *Ph. papatasi* (N = 87, 12.7%) (Table 2). 

The relative abundances within the Saddaguia region also showed the dominance of two sandfly species belonging to the *Larroussius* subgenus: *Ph. perfiliewi* and *Ph. perniciosus,* which are considered sympatric in the studied area. The relative abundance of *Ph. papatasi* was higher in Saddaguia compared to Utique. This species is most abundant in the arid zones of central and southern Tunisia. The Equitability (E) value also confirmed that the sandfly fauna is dominated by just two species at this site. It is important to note that no *Sergentomyia* species were collected at either site.

### 3.2. Morphological Analysis

**Figure 2:**
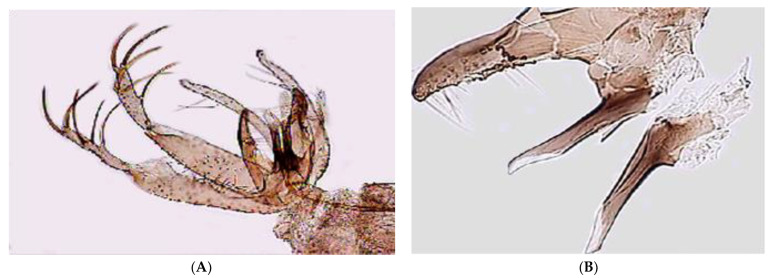
Photomicrograph of the genitalia (**A**) and parameral sheath (**B**) of *Phlebotomus perfiliewi* male.

The male of *Ph. perfiliewi* is easily distinguished by the characteristic shape of the parameral sheath (=aedeagus or penis), which has a flattened transparent apex on the ventral side and bears 4 to 5 serrated denticles on the opposite side (Figure 2A,B).

**Figure 3:**
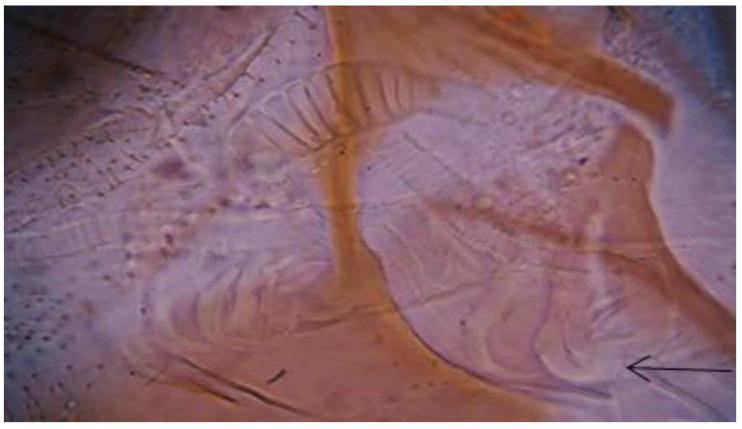
Photomicrograph of the female *Ph. perfiliewi* spermathecal duct (arrow indicates the large triangular wrinkled pocket).

The female spermatheca includes 10 to 18 rings. Their identification is mostly based on the base of their individual spermathecal ducts which open into a large triangular wrinkled pocket (Figure 3).

### 3.3. Molecular Analysis

Of the 20 specimens analyzed, only 12 sequences were obtained and deposited in the NCBI database (GenBank accession numbers are listed in Table 3). All sequences identified *Ph. perfiliewi* as the most probable species (Table 3), which fully agreed with the morphological identification.

**Figure 4:**
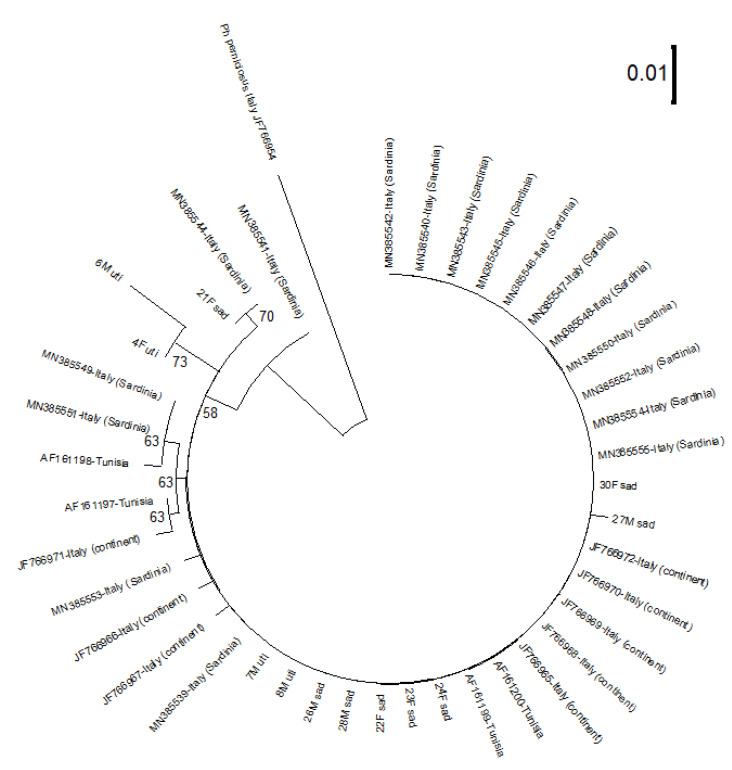
Maximum likelihood tree obtained using a Hasegawa-Kishino-Yano model on a dataset of cytochrome b mtDNA sequences. *Ph. perfiliewi* collected from the sites of Saddaguia (sad) and Utique (uti) and processed in the present study are compared with available sequences in Genbank after rooting on *Ph. perniciosus*. The tree topology was supported by 1000 Bootstrap replicates. Bootstrap values >50% indicated close to the considered nodes.

The phylogenetic tree including our 12 sequences compared to the 29 of *Ph. perfiliewi* available in GenBank, compared to 1 *Ph. perniciosus* sequence is shown in Figure 4 which also exhibits the details of the geographic origins and accession numbers.

The *Ph. perfiliewi* sequences of the processed Tunisian specimens cluster with most of the Italian ones available in the GenBank (Figure 4), except 4F uti and 6M uti which are clearly themselves individualized mostly due to 3 bp transversions, and 21F sad shared its haplotype with the Sardinian MN385544 (1 nucleotide differing from the main haplotype). All the processed *Ph. perfiliewi* are well separated from *Ph. perniciosus*. The overall nucleotide divergence within all *Ph. perfiliewi* Cyt b sequences analyzed is just 1%. 

## 4. Discussion

For a better understanding of sandfly faunistic patterns, we quantified species diversity [31] and found that diversity was low in both sites, as *Ph. perfiliewi* was the most abundant species. The morphological identification of sandfly species in this two sites revealed the presence of three species belonging to the subgenus *Larroussius* (*Ph. perfiliewi*, *Ph. pernicious*, and *Ph. longicuspis*) and a single species of the subgenus *Phlebotomus* (*Ph. papatasi*). While sampling was performed during the first peak of activity of sandflies, our findings are in agreement with those performed in the same surveyed region during the first and second peaks of activity, which all confirmed the dominance of *Ph. perfiliewi* [4,16]. Despite the bioclimatic difference between the two study sites, sandfly species composition is similar, with a predominance of *Ph. perfiliewi* in both sites.

Originally, the two investigated sites belonged to different biogeographical areas having differences in sandfly faunas [9,10,14]. *Phlebotomus perfiliewi* is the predominant sandfly species in the humid and sub-humid areas and was absent in the arid areas until 1980 [14]. Similarly, *Ph. papatasi* is absent from the northern humid area and is the predominant sandfly species of the arid and Saharan biogeographical areas [14,20,38,39]. An important extension of *Ph. perfiliewi* toward the central arid biogeographical areas occurred following environmental changes due to the development of irrigation systems in that region [4,20,40]. It is important to note that sandfly sampling procedures in studies performed before 1980 and after were made using different methods: sticky traps before 1980 and CDC light traps after. Studies performed in the central arid areas using both sandfly sampling methods showed the predominance of *Ph. perfiliewi* [4,40,41,42]. Thus, the differences observed between studies performed in the past and in the present could not be attributed to the use of different sampling methods rather than the occurrence of environmental changes. In the central arid zones, with intensive irrigation such as Saddaguia, *Ph. perfiliewi* has been established and has become the most abundant sandfly species [4,40,41,42], which has led to the emergence of ZVL in the south of the Tunisian ridge [4,5,19,20,21]. 

*Phlebotomus perniciosus* is the second most common sandfly species in both sites. *Phlebotomus perniciosus* was formerly predominant in semi-arid areas north of the Tunisian Ridge and rare in arid biogeographical areas in the south [14,24,28]. No atypical forms of *Ph. perniciosus* were observed. Aridity is considered as a limiting factor for the geographical distribution of *Ph. perniciosus* [15,28]. Entomological investigations through a north-south transect performed in 1980 and in 2006 reported relative abundances of *Ph. perniciosus* in the arid bio-climatic zone of 0.3% (*n* = 11,724) and 20% (*n* = 1024), respectively [14,20]. Therefore, the geographical distribution of *Ph. perniciosus* is extending toward arid biogeographical areas located in central Tunisia. Irrigation appears to be responsible for the pullulating of *Ph. perniciosus* in Saddaguia [4].

*Phlebotomus longicuspis* has also been found in all bio-geographical areas of Tunisia, from the humid north to the Saharan, with a predominance of 60% in the Saharan biogeographical areas [20]. Thus, the ecological plasticity of this sandfly species allows its wide geographical distribution as it can apparently survive in both humid and arid regions.

*Phlebotomus papatasi* is rare in the humid, sub-humid, and semi-arid bioclimatic zones, and becomes abundant in the arid and Saharan bioclimatic zones [14,20,38,39]. Subsequently, the geographical distribution of *Ph. papatasi* is related to bioclimatic factors [15]. Increased humidity appeared to be a limiting factor for the geographical extension of *Ph. papatasi*, as the abundance of this species increases with the aridity.

*Phlebotomus sergenti* and *Sergentomyia* spp. were absent at both sites. Since both sandfly species are known to be abundant only in villages adjacent to mountainside arid areas of central Tunisia [43], their absence within the peri-domestic areas was expected. 

The goal of the study was to characterize *Ph. perfiliewi* from two Tunisian populations. We randomly selected 20 specimens for molecular studies and we succeeded in sequencing 12 specimens. Molecular typing has confirmed the morphological identification of *Ph. perfiliewi* and also has emphasized some genetic variability. The 12 *Ph. perfiliewi* sequences collected in this study are grouped with Italian specimens. The sandfly population circulating in the site of Saddaguia is homogeneous while the population of Utique seems to be heterogeneous, especially with the specimens 6M uti and 4F uti showing variability mostly due to three variable nucleotides. Our findings provided strong evidence that the circulating populations are in fact *Ph. perfiliewi* s. st. [22]. Our results are in agreement with previous studies performed in the surveyed regions showing that *Ph. perfiliewi* is endemic in the central arid areas [16,26]. 

## 5. Conclusions

In the present study, we characterized morphologically the sandfly fauna in two distinct bioclimatic regions of Tunisia showing that *Ph. perfiliewi* is the dominant sandfly species in the humid and in the highly irrigated arid bioclimatic areas located in northern and central Tunisia, respectively. In addition, we confirmed the identification of *Ph. perfiliewi* populations with molecular markers. Our results showed complete agreement between morphological and molecular identification. Therefore, monitoring sandflies systematically is of major epidemiological importance for effectively controlling sandfly-borne diseases such as ZVL.

## Figures and Tables

**Table 1:** Biodiversity indexes and dominant sandflies species in the site of Utique.

	*Ph. perf.* 113	*Ph. pern*. 92	*Ph. long*. 20	*Ph. pap*. 8
pi	0.48497854	0;39484979	0.08583691	0.03433476
Pi^2^	0.23520419	0.15590635	0.00736798	0.00117888
∑pi^2^	0.39965739			
Ar	48.5	39.5	8.5	3.5
Is	2.50214315			
H	1.51190652			
Equitability	0.75336671			

**Table 2:** Biodiversity indexes and dominant sandflies species in the site of Saddaguia.

	*Ph. perf.* 295	*Ph. pern*. 238	*Ph. long*. 65	*Ph. pap*. 87
pi	0.43065693	0.34744526	0.09489051	0.1270073
pi^2^	0.1854654	0.12071821	0.00900421	0.01613085
∑pi^2^	0.33131866			
Ar	43.0656934	34.7445255	9.48905109	12.7007299
Is	3.01824228			
H	2.46274993			
Equitability	1.22716173			

**Table 3:** Homology (%) between *Ph. perfiliewi* samples identified in Utique and Saddaguia obtained and the available sequences from the NCBI database.

Code of Specimens (GenBank Accession Number)	Nearest Sequence	Query Cover	Homology	Identified Species
4F_uti (ON515731)	MN385555	100%	99.05%	*Ph. perfiliewi*
6M_uti (ON515732)	MN385555	100%	97.79%	*Ph. perfiliewi*
7M_uti (ON515733)	MN385555	100%	100%	*Ph. perfiliewi*
8M_uti (ON515734)	MN385555	100%	100%	*Ph. perfiliewi*
21F_sad (ON515735)	MN385544	100%	100%	*Ph. perfiliewi*
22F_sad (ON515736)	MN385555	100%	100%	*Ph. perfiliewi*
23F_sad (ON515737)	MN385555	100%	100%	*Ph. perfiliewi*
24F_sad (ON515738)	MN385555	100%	100%	*Ph. perfiliewi*
26M_sad (ON515739)	MN385555	100%	100%	*Ph. perfiliewi*
27M_sad (ON515740)	MN385555	100%	99.68%	*Ph. perfiliewi*
28M_sad (ON515741)	MN385555	100%	100%	*Ph. perfiliewi*
30F_sad (ON515742)	MN385555	100%	100%	*Ph. perfiliewi*

## Data Availability

GenBank accession numbers of specimens sequenced in the present study are ON515731-ON51574.
